# Capacity Development in an Undergraduate Nursing Program in Vietnam

**DOI:** 10.3389/fpubh.2018.00146

**Published:** 2018-05-11

**Authors:** Sunjoo Kang, Thi Thuy Trang Ho, Thi Anh Phuong Nguyen

**Affiliations:** ^1^School of Nursing, Cheju Halla University, Jeju, South Korea; ^2^Faculty of Nursing, Hue University of Medicine and Pharmacy, Hue, Vietnam

**Keywords:** capacity, development, evaluation, nursing program, Vietnam

## Abstract

**Background:** Nurses are an essential human resource to ensure a healthy population and support the socio-economic development. However, little research has focused on the capacity development of nurses.

**Objective:** The performance of a capacity development project for an undergraduate nursing program in Vietnam was reviewed to share lessons.

**Design:** A descriptive case report.

**Setting:** A baccalaureate nursing program in Vietnam from June 2014 to June 2016.

**Methods:** A case report was analyzed in terms of the project's process, and the outcomes of 2 years' activities were evaluated.

**Results:** Practice-based curriculum redesign and two basic nursing subjects were developed after five rounds of curriculum workshops. To improve application efficiency, two nursing experts were dispatched to provide instructions regarding the application of the new subjects. Three candidates were invited to complete their master's and doctoral studies in Korea. An advanced nursing education environment was supported with simulation labs equipped within a ubiquitous network. The result of experts' evaluation was excellent by every criterion of the Organization for Economic Co-operation and Development—Development Assistance Committee.

**Conclusions:** The capacity development of a nursing program was possible through ownership, accountability, and results-based management. Gradual improvement in nursing academic and clinical capacity building based on research evidence can empower partner countries' nursing leadership. Introduction.

## Introduction

Nurses are an essential human resource for strengthening the health system, as they help to ensure a healthy population, and support socio-economic development. The World Health Organization (WHO) identified six building blocks critical for a national health care system to result in improved health and effectiveness (1). The WHO's global standards for the undergraduate education of nurses include the need for a sound understanding of the determinants of health. According to Shin et al. (2), the International Council of Nurses (ICN) also identified the understanding of global healthcare challenges as a global nursing leadership outcome and guided the global standards of initial education by the WHO. These recommendations were also applied to a low-resource setting country; however, it would be very difficult to improve a health care system without human resource development. Much research on nursing program evaluation has examined general programs; however, the results of previous studies by Njie et al. (3), Koto et al. (4), Kang (5), and Kang, et al. (6) revealed that only a few studies were focused on global health or global health nursing.

The term “capacity development” in this article refers to the process by which faculty and organizations get, improve, and maintain the skills and knowledge needed for curriculum development, teaching, and guiding nursing students in keeping with the global nursing educational standards (4). The effectiveness of a nursing program as outcomes of graduates' competencies can be achieved by the development of faculty competencies and environmental capacity. Most nursing undergraduate programs involve competency building, which focuses on an individual's ability to perform activities related to work. Competence stresses the skills a person should be able to demonstrate or the general capability to carry out one's job. Ultimately, the goal of an undergraduate nursing program is to foster students' competence appropriate for the global nursing environment, as well as for their countries' clinical nursing practice. However, there are many differences in competency building between developed and developing countries, because nursing programs are affected by resource capacity and professional social position. Especially, Njie et al. (3) pointed out the importance of programs including faculty development or leadership development rather than institutional or national education reform in developed and developing countries. Furthermore, very few studies have examined long-term outcomes.

The process of curriculum design is very important because it may provide a learning opportunity to the participants on philosophy, components, and the global direction. The analysis, design, development, and implementation (ADDIE) model is a linear approach to curriculum design; therefore, it is very easy to identify the process and activities. According to Voogt et al. (7), the ADDIE model is useful for recognizing the core design activities of the curriculum design practices.

This study aimed to review a 2-year project of capacity development for an undergraduate nursing curriculum in Vietnam, to share lessons learned and to discuss strategic cooperation direction, which influences nursing education policy and health care quality in Vietnam.

## Background and rationale

### Background

Vietnam's health care system is predominantly a public-sector delivery system involving central and specialized hospitals, provincial and district hospitals, community health stations, and village health workers. This means that government regulations and policies can exert a powerful effect on the public sector. Vietnam's nursing education system has been developed over the last 20 years. There are two main programs to become a nurse: bachelor programs and 2-year programs. Four-year bachelor's programs are offered primarily in 10 national universities and three private universities; other nursing programs at the provincial level are 2-year junior college level programs.

The author first visited Hue University of Medicine and Pharmacy (HueUMP) in July 2013 to request participation in the project for capacity building of hospital nurses in Vietnam, which was sponsored by the Korea International Foundation of Healthcare. Thereafter, the nursing program capacity of HueUMP was observed more closely in February 2014. Thirty-one hospital nurses were invited to HueUMP and participated in a 1-week train the trainer program held in the last week of February 2014, which was led by Korean and Vietnamese instructors. The infrastructure for the training program included one classroom and two nursing department practice labs at HueUMP. After the project, focus group interviews with the dean and vice-dean of the Faculty of Nursing were conducted. During these interviews, they requested that the nursing curriculum development be focused on competency and advanced educational infrastructure, parallel to the global trends. Therefore, a project team was formed to submit a 4-year project proposal to the National Research Foundation of Korea on the practice and competency-based capacity building of nursing undergraduate programs, and the proposal was approved in May 2014.

### Rationale

HueUMP was established as a medical training institution and was administered by the Ministry of Health until 1993. The Ministry of Education and Training (MOET) took over supervision in 1994, and a full-time 4-year nursing program began in 2001, with 20 students enrolled per year, which increased to 150 students per year by 2015. Most of the nursing curriculum was designed based on a medical-oriented model and most of the faculty members were medical doctors. The first nursing master's program began at Hochiminh University of Medicine and Pharmacy in 2010, and the other two master's programs started in 2015 at Namdinh Nursing University and Vietnam National University in Hanoi.

In a cross-sectional survey or needs assessment conducted by Nguyen et al. (8), more than 95% of the faculty and students indicated that problem-based learning was a way to increase nursing competency through a belief in self-directed teaching and learning. An effort to identify a partner institution's need for capacity building was conducted in 2013, and after the implementation of the first project, there was a limitation of the nursing program in terms of the curriculum, faculty recruitment, and educational environment for simulated skill training. Furthermore, there is a difference in the educational readiness before entering nursing practice. According to Kang and Kim (9), this may result in an inequity in the entry level of practice and the career ladder in the nursing profession.

## Methods

### Design

This study is a descriptive case report from the capacity development of an undergraduate nursing program at HueUMP, Vietnam which was implemented through official development assistance between Vietnam and South Korea.

The setting was a baccalaureate nursing program including human resources and infrastructure of HueUMP, Vietnam between June 2014 and June 2016.

### Research subjects

The Faculty of Nursing at HueUMP consisted of 25 full-time and about 50 part-time faculty members. Systematic phases in previous development assistance projects by Hwang et al. (10), Shin et al. (2), and Kang et al. (11) were applied to the traditional process of analysis, design, development, implementation, and evaluation. In addition, the project performance in terms of curriculum redesign, faculty and environment support, and a student leadership program were analyzed.

### Data collection

**Figure 1:**
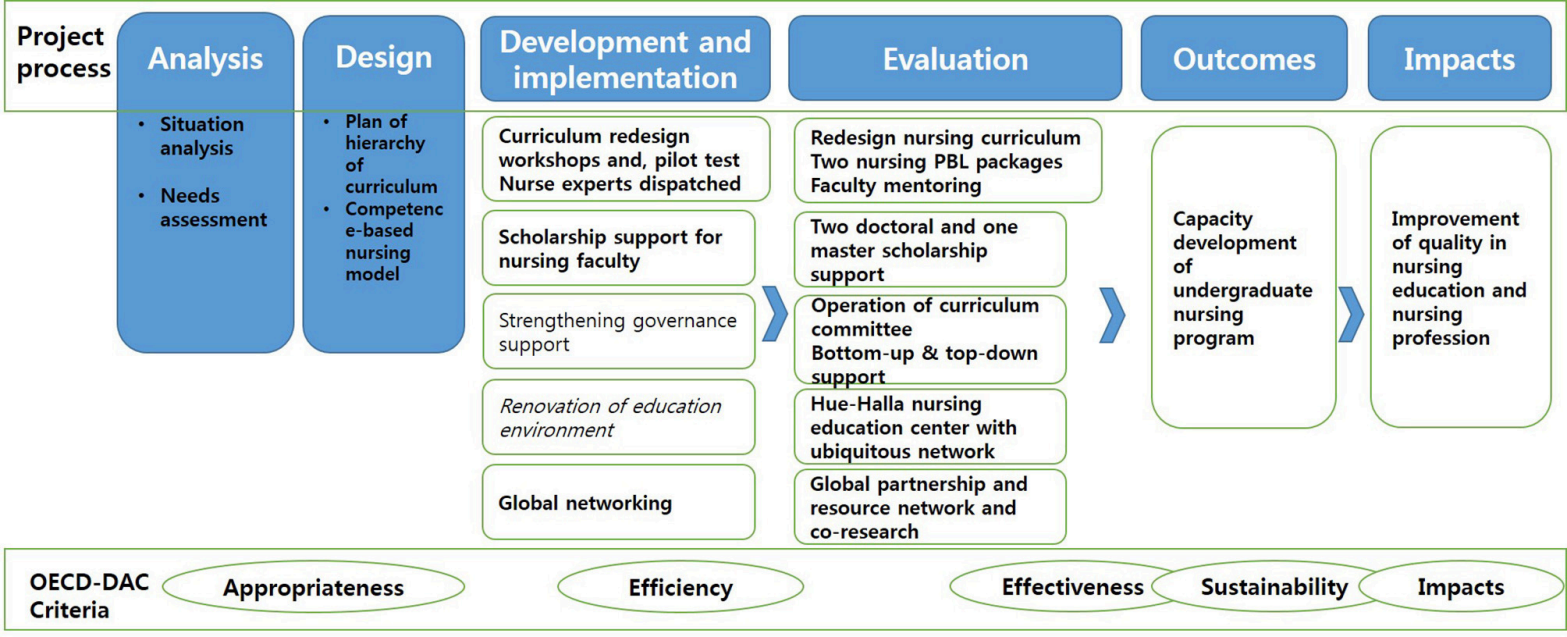
Project process.

Data were collected through the whole project process as shown in Figure 1.

In the analysis phase, situation analysis, and needs assessment were conducted three times. First, observational analysis was completed by hospital nurses in the trainer program during the last week of February. Second, in-depth interviews with deans and vice-deans of the Faculty of Nursing, HueUMP were conducted mid-April 2014. The analysis was conducted after the project launch from November to December 2014 using questionnaires.

In the design phase, capacity development of a 4-year project was designed to upgrade a current HueUMP program to a competency-based nursing program. This was the first attempt at a comprehensive project, which strengthened the overall capacity of the nursing program; the activities of the Faculty of Nursing were otherwise limited to cooperation with short-term nursing faculty training and master's programs, support for nursing books, and co-practice for patients or communities by students from Finland, America, Australia, and the Netherlands.

In the development phase, five areas of capacity development activities were identified: improvement of quality of teaching and learning with curriculum redesign, scholarship support, strengthening of the governance, construction of an advanced nursing education environment, and global network for nursing education. Throughout the development process, the researcher observed, and communicated with the participants and collected data.

In the implementation phase, curriculum development workshops and faculty capacity building sessions were held in Korea and Vietnam. Renovation of education infrastructure, activities of the curriculum development committee, and evaluation were implemented in Vietnam. Related reports and comments were collected for this study.

In the evaluation phase, the accomplishment of the first phase of the project was evaluated by the project team, using the nursing faculty's self-evaluation, followed by external nursing and medical experts' evaluation each year based on five criteria of the Development Assistance Committee of the Organization for Economic Co-operation and Development (OECD-DAC). The criteria were relevance, efficiency, effectiveness, impact, and sustainability in the guidelines for project and program evaluation by the Austrian Development Cooperation (12). Those evaluation reports and seminar comments were used for this study.

### Data analysis

All the project activities' outputs and 2 years' performance of projects were analyzed by areas of capacity development aligning with OECD-DAC evaluation criteria as shown in Figure 1 and described below.

## Results

### Activities for improving quality of teaching and learning

Three invited training sessions for curriculum development were conducted with delegates of the Faculty of Nursing, HueUMP, and two visiting workshops by Korean nursing education experts at Cheju Halla University were conducted to expand faculty members' ability to redesign nursing curriculum, during summer and winter vacation in 2014 and 2016, to avoid routine academic program operations as shown in Table 1. A nursing curriculum committee was formed at HueUMP, and this committee advised the subject adjustments for two newly developed basic nursing packages of three credits each in the formal curriculum. However, there was a limitation of the nursing program redesign, because the MOET set an official framework curriculum for the national level and only 20% of the total credits were under the control of the university itself.

**Table 1:** Curriculum development workshops.

	**Topic**	**Place**	**Workshop method**	**Dates**	**Special lecturer**	**Participants**
1st	Development process of nursing curriculum	CHU^*^, South Korea	Action Planning	22–24 Oct 2014	Chair, Dept. of Nursing, CHU^*^ Dean, Faculty of Nursing, HUMP^**^	9 delegates from faculty of nursing, HUMP^**^
2nd	Theoretical base of nursing curriculum and the case of Mongolia	CHU^*^, South Korea	Curriculum designing	4–6 Dec 2014	Dean, Ulanbaataar University, Mongolia	9 delegates from faculty of nursing, HUMP^**^
3rd	Creative thinking in nursing education	HUMP, Vietnam	Curriculum designing	27–29 Jan 2015	Dean, Ulanbaataar University, Mongolia	All members in Faculty of Nursing, HUMP^**^
4th	How to develop a problem-based nursing subject(package)	CHU^*^, South Korea	Preparation of instructional material	3–5 Dec 2015	Director Halla-New Castle PBL Center, CHU^*^	9 delegates from faculty of nursing, HUMP^**^
5th	Current nursing research	HUMP^**^, Vietnam	Micro teaching	19–21 Jan 2016	Professor Kim, Yonsei University	All members in Faculty of Nursing, HUMP
6th	Outcome sharing workshop	Hoian Hotel Vietnam	Present outcomes	24 Mar 2016	Project team of HUMP, and Korean advisors dispatched to HUMP^**^	Project team Guests from America

* *CHU, Cheju Halla University*;

** *HUMP, Hue University of Medicine and Pharmacy*.

To monitor and provide active support onsite during the nursing program renovation and faculty capacity building, a retired nursing advisor was dispatched to encourage curriculum development committee activities. In addition, a retired nursing professor with profound experience in problem-based learning method and curriculum development was dispatched to mentor the application of new nursing subjects in problem-based learning packages and pilot testing with nursing students, at HueUMP as shown in Table 2.

**Table 2:** Mentor and advisor's role in redesigning the nursing curriculum.

	**Qualification**	**Role**	**Duration**	**Funding support**
Advisor	Experiences in nursing administration and education over 30 years	Coordination of project team	June 2015–June 2016	National Research Foundation of Korea
Mentor	Experiences in nursing education especially applying problem-based learning over 30 years	Mentor for developing and applying new nursing subjects	Dec 2015–Mar 2016	Project team

For faculty training, several Korean nursing education experts' invited workshops were conducted to increase the quality of teaching and learning, such as creative thinking in nursing education, development of two problem-based learning packages, nursing statistics, and writing for research journals in English.

### Activities for scholarship support for nursing faculty

The most critical problem for the undergraduate program was lack of faculty with a nursing background. From a long-term perspective, fostering faculty human resources is the most important capacity building aim at the personal and institutional level. Although a tripartite collaboration for Vietnam, with master's and doctoral programs in Thailand and support for tuition from the Korean government, was considered, there were many related political issues.

In terms of the nursing faculty, clinical practice assistants were nurse graduates waiting for sponsorship for their master's and doctoral study, so the project team had a meeting and asked for funding from the Ministry of Education of Korea. Finally, at the request of HueUMP, three candidates were selected for 1 year of Korean language support and master's and doctoral nursing programs in Korea. All tuition was sponsored by the Global Korea Scholarship program under the Ministry of Education in Korea.

After 1 year of Korean language education and approval of the qualification of proficiency in the Korean language, the candidates could enter the advanced nursing program in Korea. The first three candidates came to Korea in 2016 and started language training in the designated Korean language center.

### Activities for strengthening governance and administration

The curriculum redesign process should include the needs of nursing consumers (such as hospitals' request to educate nursing students appropriate to the current clinical environment, patient needs, advancement in medical techniques, etc.) and internal consensus among faculty members, university authorities, and the MOET in Vietnam. In total, 20% of the nursing program was managed by the university authorities without the approval of the MOET. A nursing curriculum redesign committee was organized with 15 members: two external experts in clinical nursing, two nursing lecturers with nursing backgrounds, four nursing faculty and five university authorities, and two Korean nursing professors from the project. This committee met at least bimonthly to reach a consensus on the revised nursing curriculum. All of the invited training faculty in Korea were selected based on the criteria of gender and educational background in medicine or nursing on an equal basis.

Through the project process, a bottom-up approach for new nursing subject development and top-down approach for policy support in the university and nationwide were reciprocally applied. All of the purchased educational equipment and the remodeled Hue-Halla nursing education center were managed by the project team, with the support of the University President, which was acknowledged in a national economics magazine.

### Renovation of an advanced nursing education environment

Based on a needs assessment and the study result of Kang et al. (11), a learner-directed teaching method was considered, and finally, a problem-based learning approach was selected as the method for newly developed nursing subjects. In addition, HueUMP considered taking the lead position in advanced nursing simulations as well as emergency medicine education in Vietnam. Finally, the blueprint of the nursing education center was approved by the Rector Board of HueUMP in December 2014 to secure a proper educational environment and promote the effects of education. Problem-based learning and teaching strategies were applied to learning with over 200 nursing students, which showed positive effectiveness (8) (see Table 3).

**Table 3:** Number of nursing students attending workshops for problem-based learning strategies.

**Year of nursing education**	**Number of students**
2nd	74
3rd	109
4th	54
Total	236

Renovation of the nursing education environment was coordinated through mutual communication and based on agreement of projects. This included two nursing simulation training labs and three labs for basic life support and advanced life support training. In addition, a two-way computerized discussion room with a ubiquitous learning network was furnished on the fifth floor of the newly built learning resource center in 2015 with 355 m^2^ of space. This new building was constructed through funds from an American non-government organization called Atlantic Philanthropies. The discussion room was appropriate for small group teaching and learning using five desktops for group tables and one instructor control table. All of the learning labs and spaces were networked for ubiquitous learning and an inauguration ceremony of the Hue-Halla center was held in September 2015 after several months of pilot operation in each lab and space.

### Sustainable global network in nursing education

To meet the future nursing leaders and potential faculty candidates, a special student program was developed through an educational needs assessment. In the needs assessment study by Kang et al. (13), Borich's needs assessment tool was used to measure participants' perceptions of present and required competency levels to identify the educational needs of an undergraduate nursing educational program. After students' needs assessment among Korean, American, and Vietnamese students, this program was designed, revised, and implemented. This program was operated for 1 week at Hanoi and Hue city in 2015 with 30 Korean and Vietnamese students, respectively, and the effects of this program were analyzed by Kang et al. (6). The results were prominent for knowledge change in the healthcare system, function of the United Nations and WHO, and attitude change in cultural and global leadership. These global partnership programs were theoretically based on Wilson et al.'s (14) work on global health competencies.

## Discussion

Nurses in low-resource setting countries can be empowered when their basic educational capacity development and the culture of nursing practices become evidence-based. According to Shin et al. (2), the ICN fosters global nursing leaders and, through uniform and standardized efforts, strives to assist in improving nurses' social position to contribute to people's well-being. Since the first announcement of educational standards in the Goldmark report in 1923, the importance of standards in nursing programs has been publicly discussed and efforts have been made by the member nurses of the ICN.

Curriculum redesign and development is a strategic approach because it encompasses and meets global nursing education norms as well as human resource development and infrastructure refurbishment. According to Evans et al. (15), Kang et al. (6), and Shin et al. (16), the effect of the redesigned curriculum takes more than 4 years as the graduates gradually apply the new nursing curriculum. In most research programs, faculty training was approached in two ways: one for present faculty and the other for faculty candidates. Therefore, a review of the effect of applying two basic nursing problem-based learning packages will be considered this year after analysis of pilot testing in 2016.

In this study, the main areas of faculty training in previous research were upgrading knowledge and skills, teaching method, transforming theory into practice, research ability, leadership, and clinical education capacity as demonstrated in previous studies (3, 4, 6). Throughout this project, nursing curriculum redesign was focused on competency improvement of nursing graduates and problem-based learning as a new teaching method as demonstrated by Ghalenoei et al. (17). It can be concluded that faculty capacity building should be designed in two directions for present faculty members and potential candidates as nursing graduates. Despite the importance of scholarship training master's and doctoral studies for faculty candidates, mentioned by Njie et al. (3), sustained financial support is needed because most candidates in developing countries cannot afford tuition for 2 or 3 years; therefore, they wait years to receive sponsorship. In particular, the lack of doctoral programs in developing countries itself delays the revival of nursing education and professional advancement. In this regard, long-term collaboration with master's programs for faculty in developing countries contributed to nursing leadership capacity and health policy engagement. Another consideration is a tripartite collaboration because there is a culture of friendly developed neighboring countries surrounding project partners from developing countries. Therefore, it would improve efficiency to dispatch more candidates to neighboring countries, which offer advanced programs, rather than invite master's or doctoral candidates to Western developed countries. However, potential political conflict between the project team and financial support institution is expected.

Regarding ownership and accountability, all the faculty members were welcome to contribute ideas and opinions about modules or nursing subject development and the application process. Another method of change governance was to set criteria to form a committee based on major and gender, to avoid male and non-nursing major dominant situations. However, there has been very little research on governance or administration training for nurses; it was good for capacity building through bottom-up and participatory approaches in project implementation. Koto et al. (4) and Shin et al. (16) highlighted the role of a self-governance body in a partner country as a way of bottom-up decision making. Though the formation of a nursing curriculum committee of this project was top-down rather than bottom-up, the members were very energetic and enthusiastic in making changes adopting problem-based teaching and learning methods.

In terms of educational environment, Evans et al. (15) also indicated the importance of infrastructure improvement parallel to faculty development. This project did approach what Evans emphasized. In addition to environment renovation, the assistance of application of new nursing subjects through co-stay with the Vietnam project team was a success factor of this project. The Faculty of nursing, HueUMP mentioned the help of two Korean mentors in the development and application of new nursing subjects and that it was their first experience of the 15 years of global partnership with other countries' nursing experts. However, they were only invited or visited to show or teach new nursing research or teaching methods.

Global partnership is also another success factor for future sustainability; however, it was hard to identify networking with other countries in the present project activities. The majority of nursing programs in HueUMP have partner institutions worldwide, including other less-developed countries, and it is not difficult to meet global partners in those developing countries. According to Sies et al. (18), Tvelt et al. (19), and Lasater et al. (20), the respect of partner nursing program's partners may enable sustainable performance and create a future in nursing as a global network. It would be a very good strategy to use witnesses outside of the project to monitor and global partners to encourage faculty's efforts in upgrading nursing programs from various viewpoints and cooperating as required with outside experts on weak areas. Regarding student leadership programs for guiding visionaries in the nursing profession, there were several student-partnership programs before this project, based on a project review of Kang et al. (11). However, Vietnam and Korean students increased their creative thinking and nursing leadership through this project more than in the past (6).

This case report on a Vietnam nursing capacity building project was very similar to the situation of the Republic of Korea. The Republic of Korea was a very poor country in the 1950s after the Korean War, and Western assistance for socio-economic development helped to revive Korean society, especially with respect to scholarship programs. In an effort to improve human resource development, the first nursing doctoral program began in 1973 and changed to an advanced nursing curriculum model from a medical-oriented program after there were enough faculty members with the required qualifications to operate the program during the 1960s and 1970s. In terms of war and development processes, Korea's past experience could be a good model for Vietnam's advancement in all areas. Vietnam has seen change in its socio-economic level since the Doi-moi economic reform in 1986 and the Vietnam War in the 1970s. Though Vietnamese people possess positive potential, a fundamental shortage of human resource development and know-how for further advancement in higher education means that long-term cooperation in education is needed.

Though the nursing profession usually lacks firm and powerful strategic representation in healthcare policy, according to Kang and Kim (9), in developing countries, academic and clinical competencies should be advanced in parallel with evidence-generating research works. National nurses' license examinations, self-regulation through nursing ethics, and supplementary training to maintain current nursing science can guarantee nursing positions firmly rooted in the fourth-industrial revolution age.

## Conclusions

According to the evaluation of each criterion, project teams showed good communication with stakeholders in Vietnam and Korea and accomplished all of the performance indicators within two project years with the full use of the budget. The appropriateness of the project was guaranteed by Vietnam's socio-economic development plan, HueUMP's strategy of development, and Korea's high education know-how transfer vision. In particular, the project outcome was remarkable for resource networking through understanding and cooperation with global partners visiting the Hue region. The impact of the project was publicly promoted naturally to North Vietnam, and one faculty member visited HueUMP to witness what was achieved. To guarantee sustainability, the inclusion of educational policy or regulations and sharing project outcomes with faculties in other regions should be considered.

However, there is a gap among healthcare professionals in their educational level and national standards, self-regulation, social position, difference between academic and clinical reality, and evidence-producing research ability. In developed countries, healthcare professionals have come to be recognized as a healthcare team and respect other professional fields; however, this culture is lacking in developing countries. Therefore, it takes time to increase the level of the nursing profession as a healthcare team in terms of educational and clinical capacity, based on the linkage of those capacity areas to research evidence.

In conclusion, the priority success factor of project accomplishment was ownership and accountability of the partner institution or recipient organization. A nursing program in developed countries would not be appropriate to apply directly to developing countries, because of differences in culture, educational development level, and preparing faculty. Further research should consider trans-cultural and participatory approaches for sustainable development.

## Author contributions

SK substantially contributed to the study conception, design, and writing of the manuscript, as well as acquisition and interpretation of the data; TN contributed to the study design and writing of the manuscript; TH contributed to the acquisition and interpretation of the data.

### Conflict of interest statement

The authors declare that the research was conducted in the absence of any commercial or financial relationships that could be construed as a potential conflict of interest.
